# CO_2_ Activation Within a Superalkali-Doped Fullerene

**DOI:** 10.3389/fchem.2021.712960

**Published:** 2021-07-14

**Authors:** Giovanni Meloni, Andrea Giustini, Heejune Park

**Affiliations:** ^1^ Department of Chemistry, University of San Francisco, San Francisco, CA, United States; ^2^ Department of Physical and Chemical Sciences, Università degli Studi de L’Aquila, L’Aquila, Italy

**Keywords:** CO_2_ activation, superalkali, endofullerene, ionization energy, solvation energy

## Abstract

With the aim of finding a suitable synthesizable superalkali species, using the B3LYP/6-31G* density functional level of theory we provide results for the interaction between the buckminsterfullerene C_60_ and the superalkali Li_3_F_2_. We show that this endofullerene is stable and provides a closed environment in which the superalkali can exist and interact with CO_2_. It is worthwhile to mention that the optimized Li_3_F_2_ structure inside C_60_ is not the most stable C_2v_ isomer found for the “free” superalkali but the D_3h_ geometry. The binding energy at 0 K between C_60_ and Li_3_F_2_ (D_3h_) is computed to be 119 kJ mol^−1^. Once CO_2_ is introduced in the endofullerene, it is activated, and the $\hat{OCO}$ angle is bent to 132^°^. This activation does not follow the previously studied CO_2_ reduction by an electron transfer process from the superalkali, but it is rather an actual reaction where a F (from Li_3_F_2_) atom is bonded to the CO_2_. From a thermodynamic analysis, both CO_2_ and the encapsulated [Li_3_F_2_⋅CO_2_] are destabilized in C_60_ with solvation energies at 0 K of 147 and < −965 kJ mol^−1^, respectively.

## Introduction

In 1985, Kroto and co-workers discovered an extremely stable cluster consisting of 60 carbon atoms during a study of long-chain carbon molecules. (Kroto et al., 1985). This cluster, called fullerene, has a football shape with 12 pentagonal and 20 hexagonal rings. (Kroto et al., 1985). Shortly after, a study presenting successful formation of fullerenes with a lanthanum atom trapped in the cavity of C_60_, called endofullerene, C_60_La, was published. (Heath et al., 1985). Another consequent experiment proved the stability of C_60_La^+^ against H_2_, O_2_, NO, and NH_3_. (Weiss et al., 1988). This suggested that the lanthanum atom can be “protected” by being encapsulated in the fullerene. Since then, a number of studies focusing on novel properties of C_60_ and its interactions with other species have been carried out. (Ruoff et al., 1993; Diederich and Gómez-López, 1999; Bakry et al., 2007; Mignolet et al., 2013; Wang et al., 2014; Elliott et al., 2018; Kouřil et al., 2018) Some investigations concentrated on medical applications, hydrogen storage, and various endofullerene. (Wang et al., 2013; Srivastava et al., 2016; Srivastava et al., 2017; Elliott et al., 2018; Kouřil et al., 2018). Due to the fullerene's unique cage-like cavity, a procedure called molecular surgery can be performed to entrap an atom or molecule. (Murata et al., 2006; Krachmalnicoff et al., 2016). Utilizing this technique, encapsulation of molecular hydrogen and HF were successfully achieved. (Murata et al., 2006; Krachmalnicoff et al., 2016). In addition, a recent paper by Jana and Chattaraj (2020) describes the effects of a molecular reaction environment, dodecahedrane, on the He dimer bonding. Also, theoretical studies of a new type of endofullerenes with superalkali have been performed. (Srivastava et al., 2016; Srivastava et al., 2017) Superalkalis are clusters with very low adiabatic ionization energies. (Gutsev and Boldyrev, 1982; Gutsev and Boldyrev, 1983; Gutsev and Boldyrev, 1987; Lia et al., 1988; Gutsev and Boldyrev, 1990; Tong et al., 2013a; Tong et al., 2013b). The first and most common superalkalis have the formula M_k+1_L, where M is an alkali atom with valence k and L is an electronegative atom. (Gutsev and Boldyrev, 1985; Gutsev and Boldyrev, 1987; Zhao et al., 2017). Nambiar et al. (2021) showed the importance of these compounds through a density functional computational study to improve the efficacy of redox reactions.

The concentration of carbon dioxide (CO_2_) in the atmosphere has been increasing constantly since the early 20^th^ century with rapid industrialization. (D’Amato and Akdis, 2020; Kuzovkin and Semenov, 2020). This is a globally recognized issue as CO_2_ contributes significantly to the greenhouse effect and the acidification of the oceans. (Crowley and Berner, 2001; Hönisch et al., 2012). Increment of atmospheric temperature causes climate change that threatens the overall ecosystem. To capture CO_2_ molecules present in the air, various methods have been employed such as packed column of monoethanolamine and metal-organic frameworks. (Lv et al., 2015; Li et al., 2019; Li et al., 2020). The next step is activating CO_2_ molecules and converting them to value-added chemicals such as hydrocarbon fuels. (Hu et al., 2013). The activation of CO_2_ is extremely complex due to its stability and much efforts have been made by researchers to directly convert it into liquid hydrocarbons, useful for the aviation sector as novel jet fuels (Boreriboon et al., 2018; Vogt et al., 2019; Yao et al., 2020) or into oxygenates, such as ethanol. (Song et al., 2016; Bai et al., 2017; Wang et al., 2018). This conversion, whether it involves a direct CO_2_ hydrogenation route or not, entails the usage of metal-based catalysts to ensure an overall reasonable efficiency. (Yao et al., 2020).

In our previous studies, successful activation of CO_2_ with a superalkali species, Li_3_F_2_, were presented. (Park and Meloni, 2017). The computational study showed charge transfer from Li_3_F_2_ to CO_2_, which indicates migration of the unpaired electron from Li_3_F_2_ to CO_2_. The activated CO_2_ showed geometric change such as bent $\hat{OCO}$ angle. The activated CO_2_ then can be transformed to other organic molecules with catalysis. (Liu et al., 2016; Luc et al., 2017). Removing the unpaired electron from [Li_3_F_2_⋅CO_2_] cluster weakens the interaction between Li_3_F_2_ and CO_2_ and geometry of CO_2_ returns back to the linear form. (Park and Meloni, 2017). The superalkali Li_3_F_2_ was observed and characterized experimentally. (Yokoyama et al., 2000; Haketa et al., 2002). They also confirmed three stable Li_3_F_2_ structures through a computational density functional approach. (Haketa et al., 2002). In this investigation, the Li_3_F_2_-doped fullerene and its endo-reaction with CO_2_ has been characterized using the B3LYP/6-31G* level of theory. These results are explained in terms of energetics and molecular orbitals of the species involved. In addition, these findings will be beneficial in providing insights for CO_2_ reduction and in helping the exploration of new materials with tailored properties.

### Computational Methods

Geometries and total electronic energies of the investigated species were calculated at the B3LYP/6-31G* level of theory (Becke, 1988; Lee et al., 1988) using the computational software Gaussian09. (Frisch et al., 2016). B3LYP is one of the most commonly used density functional theory (DFT) methods that employs a three-parameter exchange functional developed by Becke (1992) and Becke (1993) with a correlational functional proposed by Lee, Yang, and Parr (LYP) Becke (1988) to approximate the exchange-correlation energy. The B3LYP/6-31G* level has been employed to study endofullerene systems because it yields reliable geometries and energies. (Wang et al., 2013; Srivastava et al., 2016; Srivastava et al., 2017). Partial atomic charges are calculated based on the Mulliken population analysis (Mulliken, 1955) and natural bond orbital (NBO) population analysis. (Reed et al., 1985).

The adiabatic ionization energy (AIE) is calculated by taking the zero-point energy corrected electronic energy difference between the optimized neutral and cation, whereas the adiabatic electron affinity (AEA) is obtained by subtracting the zero-point-energy corrected electronic energy of the optimized anion and neutral. All the optimized structures have real vibrational frequencies and their Cartesian coordinates have been reported in the Supplementary Material.

## Results and Discussion

The main intent of this computational investigation is to study the interactions relevant to the reduction of CO_2_ by the superalkali Li_3_F_2_ inside our molecular reaction vessel, i.e., C_60_, and see how this environment affects the CO_2_ activation. The system is fairly large and, therefore, computationally challenging to investigate. We have analyzed the possible interactions between the fullerene and the two reactants, CO_2_ and Li_3_F_2_. All the computed energetics are reported in Table 1 together with the available literature (experimental and computed) values.

**TABLE 1:** Energetics of all the species relevant to this study calculated at the B3LYP/6-31G* level of theory. The zero-point-energy corrected total electronic energy (E_0_) is in Hartree, the adiabatic ionization energy (AIE) and adiabatic electron affinity are in eV, and the binding energy at 0 K is in kJ mol^−1^. [SA⋅CO_2_] stays for the endo superalkali⋅CO_2_ complex.

Species	E_0_	AIE	AIE liter	AEA	AEA liter	BE	BE liter
**CO**_**2**_	−188.569349	13.6	13.78 Herzberg (1966)	−1.27	−0.60 Wang et al. (1988)	−	−
					−1.60 de Vries et al. (1992)		
**Li**_**3**_ **F**_**2**_ **(C**_**2v**_ **)**	−222.473494	3.91	3.80 Hartman and Hisatsune (1966)	0.63	0.59 Hartman and Hisatsune (1966)	−	−
**Li**_**3**_ **F**_**2**_ **(D**_**3h**_ **)**	−222.459703	4.24	3.86 Hartman and Hisatsune (1966)	0.36	0.75 Hartman and Hisatsune (1966)	−	−
**C**_**60**_	−2285.799198	7.08	7.54 Sikorska and Gaston (2020)	2.25	2.68 Knapp et al. (1986)	−	−
**Li**_**3**_ **F**_**2**_ **(C**_**2v**_ **)⋅CO**_**2**_	−411.112817	5.42	5.21 Park and Meloni, (2017)	0.97	0.36 Park and Meloni (2017)	184	163 Park and Meloni (2017)
**Li**_**3**_ **F**_**2**_ **(D**_**3h**_ **)⋅CO**_**2**_	−411.096882	5.25	−	0.80	−	178	−
**C**_**60**_ **⋅CO**_**2**_	−2474.312373	7.08	−	2.27	−	−147^a^	−
**C**_**60**_ **⋅Li**_**3**_ **F**_**2**_ **(D**_**3h**_ **)**	−2508.304346	5.64	−	2.45	−	119^a^	−
**C**_**60**_ **⋅[SA⋅CO**_**2**_ **]**	−2696.601627	5.73	−	2.56	−	^a^	−

a This binding energy at 0 K corresponds to the negative solvation energy at 0 K that C_60_ exerts on the encapsulated species, reactants, Li_3_F_2_ and CO_2_, and product SA⋅CO_2_ (see text).

**FIGURE 1:**
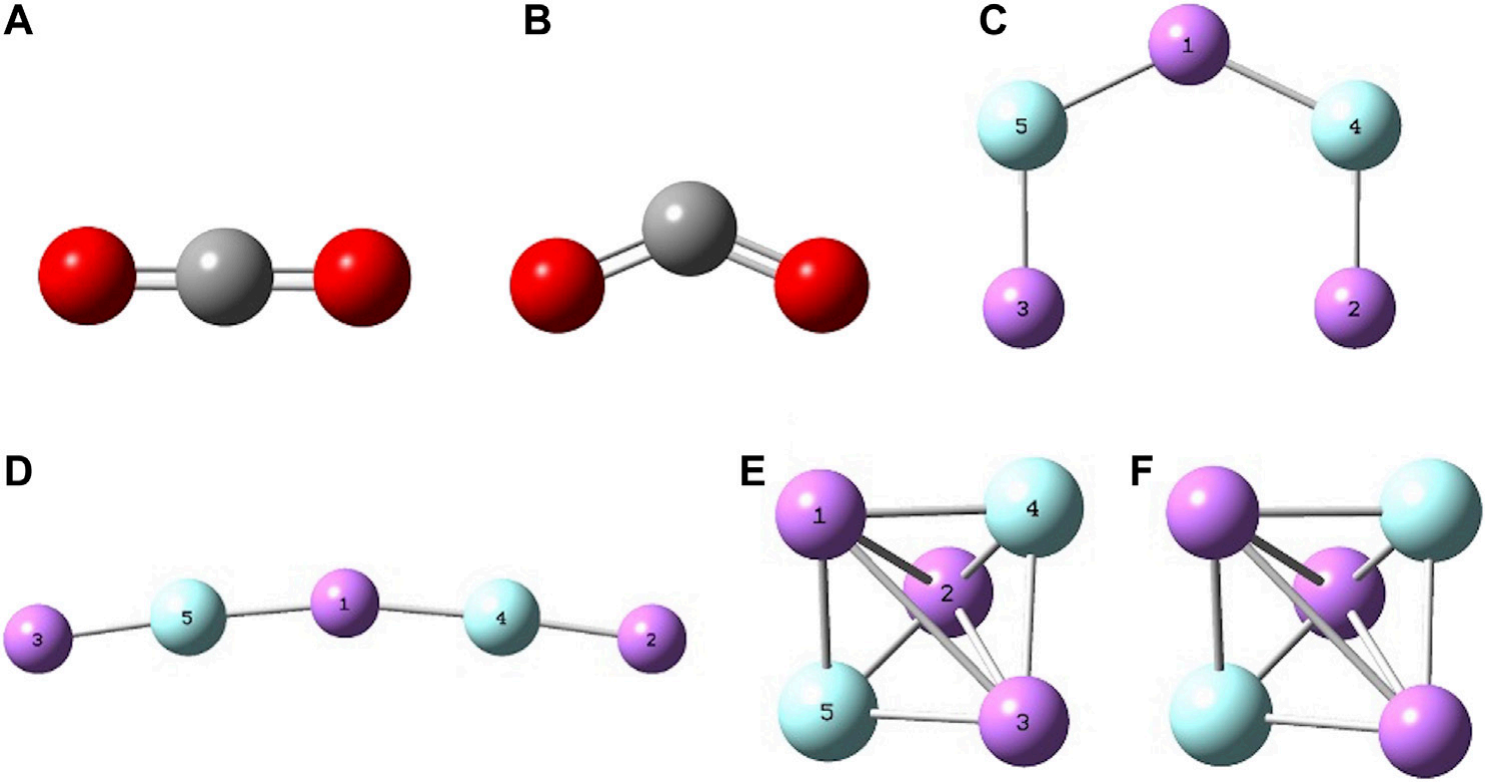
B3LYP/6-31G* optimized geometries of **(A)** CO_2_, **(B)** CO_2_^−^, **(C)** Li_3_F_2_(C_2v_), **(D)** Li_3_F_2_^+^(C_2v_), **(E)** Li_3_F_2_(D_3h_), **(F)** Li_3_F_2_^+^(D_3h_).

**FIGURE 2:**
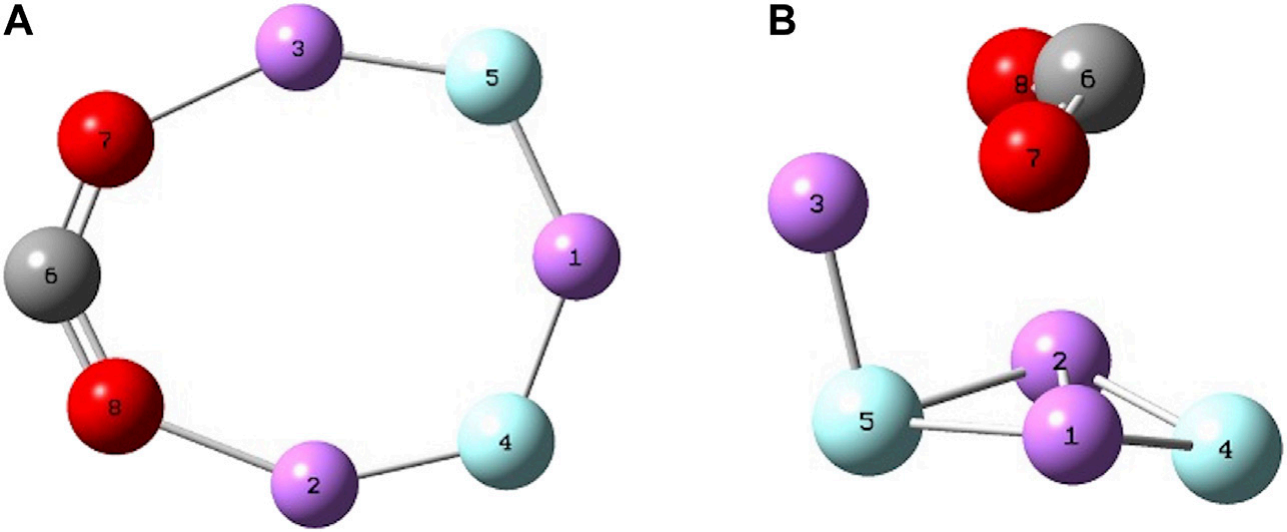
B3LYP/6-31G* optimized geometries of **(A)** Li_3_F_2_(C_2v_), and **(B)** Li_3_F_2_(D_3h_) reducing CO_2_.

Figure 1 reports the optimized geometries for CO_2_ and CO_2_^−^, Li_3_F_2_(C_2v_), Li_3_F_2_(D_3h_) and their cations. The structures of CO_2_ and CO_2_^−^ reproduce well the literature experimental values for both bond distances and bond angles. In fact, for CO_2_ we have r_C-O_ = 1.17 Å (1.16 Å) (Herzberg, 1966) and for CO_2_^−^ we have r_C-O_ = 1.25 Å (1.25 Å) (Hartman and Hisatsune, 1966) and ∠OCO^°^ = 134^°^ (127 ± 7^°^) . (Hartman and Hisatsune, 1966). Both the geometries of the lowest energy Li_3_F_2_(C_2v_) isomer, trigonal bipyramidal Li_3_F_2_(D_3h_), and their cations are in agreement with our previous work. (Park and Meloni, 2017; Cochran and Meloni, 2014). Figure 2 shows the two superalkali isomers reducing the CO_2_. The geometry for the previously studied C_2v_ isomer interacting with CO_2_ is in agreement with our previous results, (Park and Meloni, 2017), whereas the D_3h_⋅CO_2_ species is reported for the first time. When the D_3h_ structure reacts with CO_2_, the trigonal bipyramidal geometry is distorted by increasing two “equatorial” Li-Li distances, maintaining only the Li(1)-Li(2) distance of 2.30 Å, with Li(3) being closer to the CO_2_, Li(3)-O(7) = Li(3)-O(8) = 2.03 Å and increasing the axial F-F distance from 2.40 to 2.68 Å. The ∠OCO^°^ bond angle is 128^°^ and the C-O bond length is 1.26 Å. The Li_3_F_2_ isomers have similar binding energy with CO_2_, with the C_2v_ isomer presenting a stronger interaction of 184 kJ mol^−1^. These clusters can be defined as “free” or “naked” because they are isolated in the gas phase. The presented energy values are calculated at the B3LYP/6-31G* level and are within 10% from the literature reported quantities, whether they are experimental or computed at very high level of theory.

**FIGURE 3:**
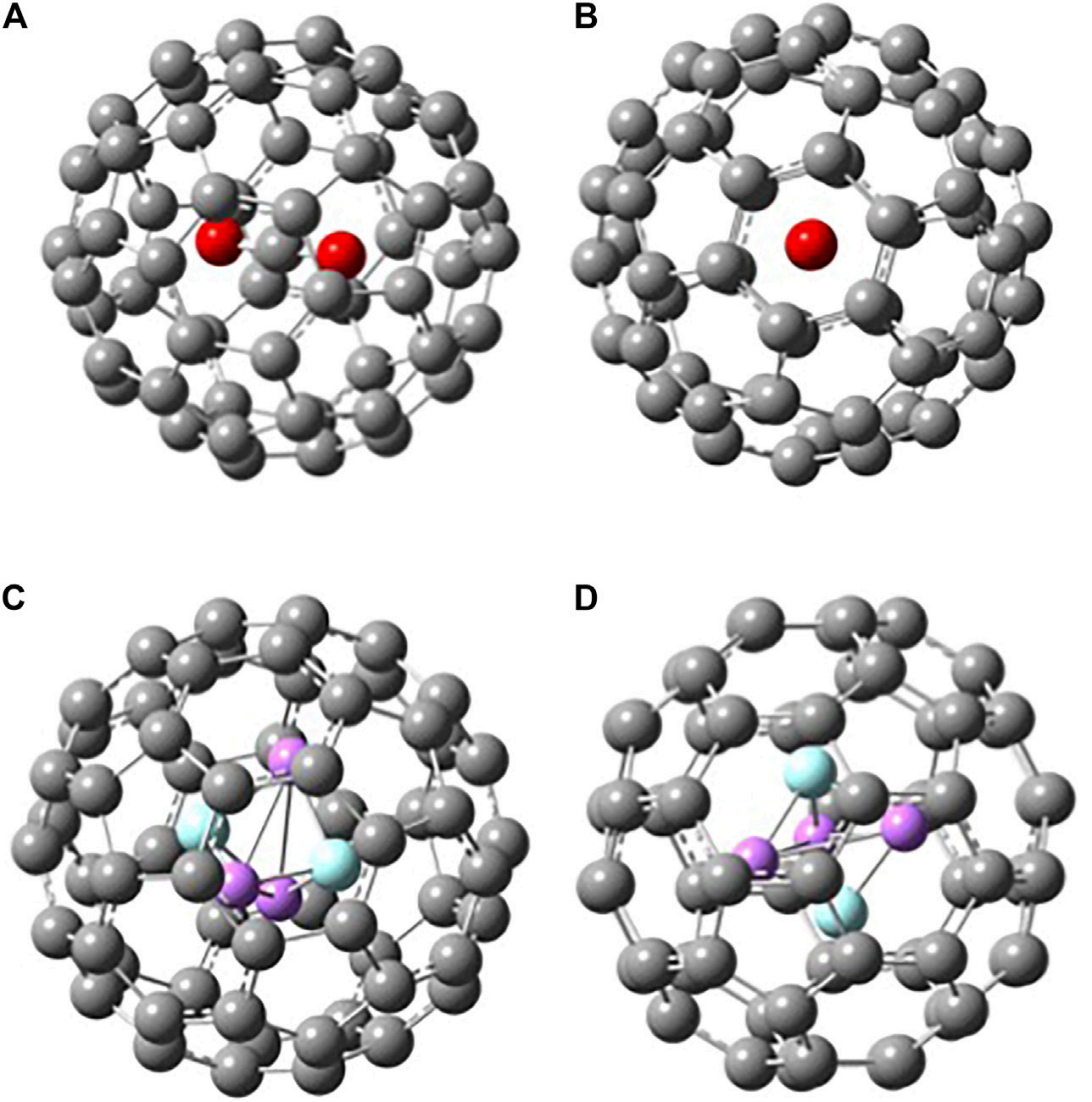
Two different views of B3LYP/6-31G* optimized structures of **(A)**-**(B)** C_60_ · CO_2_ and **(C)**-**(D)** C_60_ · Li_3_F_2_(D_3h_).

**FIGURE 4:**
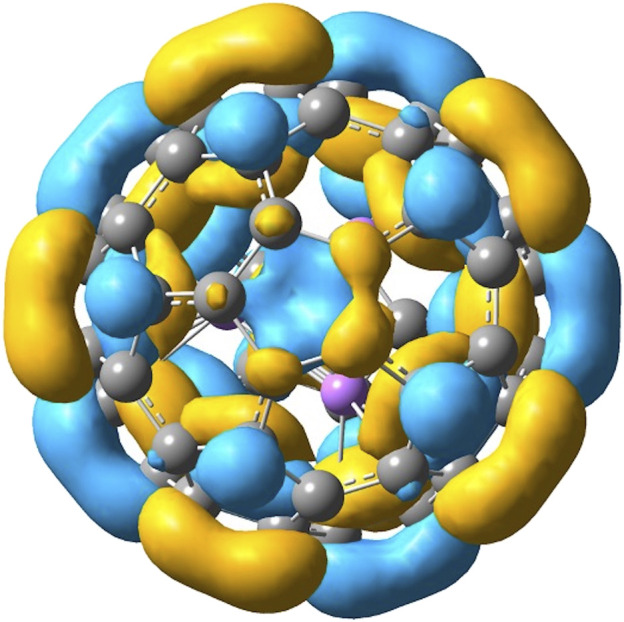
HOMO of C_60_ · Li_3_F_2_(D_3h_).

When a molecule is inserted in the fullerene (yielding an endofullerene), the chemical system is not free, but it will be subjected to the interactions with the carbon cage (“solvation effects”). In Figure 3, the two endofullerenes with CO_2_ and Li_3_F_2_ are shown. In the case of carbon dioxide, it is clear from the energetics presented in Table 1 that CO_2_ is destabilized by C_60_ having a negative binding energy at 0 K of −147 kJ mol^−1^ or a solvation energy at 0 K of 147 kJ mol^−1^, calculated as E_0_(CO_2_) + E_0_(C_60_)—E_0_(C_60_⋅CO_2_). The CO_2_ occupies the center of the C_60_, aligned with the C_3_ axis passing through a hexagonal face, minimizing its interactions with the C cage. The solvation energy is more properly defined as the Gibbs free energy change associated with the transfer of a molecule from the gas phase into a solvent, i.e., it provides the relative equilibrium populations of a species between gas phase and the solvent. Therefore, we should also know the entropy change connected with this process. The values that we are reporting in this investigation are at 0 K, so that $\Delta_{solv}H_{0}^{o}=\Delta_{solv}G_{0}^{o}=-\;BE\left(solvent-species\right)$, from which we can see that negative binding energies correspond to positive solvation energies (destabilizing effect). For the encapsulated superalkali two main findings can be noticed. First, the superalkali inside the fullerene is “forced” to assume a D_3h_ geometry, a structure almost identical to the free D_3h_ cluster but less stable than the free C_2v_ cluster. Despite having started the Li_3_F_2_ geometry optimization from different initial configurations, the optimized structure inside the fullerene resulted in the trigonal bipyramidal geometry. The Li-F distances are shortened in C_60_ from 1.83 (free superalkali) to 1.77 Å, which corresponds to a compression along the F-F distance from 2.40 to 2.21 Å, and the two Li-Li bonds elongate to 2.40 Å. The second result is that C_60_ interacts strongly with Li_3_F_2_ with a binding energy at 0 K of 119 kJ mol^−1^ or solvation energy at 0 K of −119 kJ mol^−1^, calculated as E_0_(Li_3_F_2_(D_3h_)) + E_0_(C_60_)—E_0_(C_60_⋅Li_3_F_2_(D_3h_)). This interaction is not a reduction of C_60_, where the electron from the superalkali is transferred to the fullerene. In fact, upon ionization of C_60_·Li_3_F_2_, the encapsulated Li_3_F_2_ retains its trigonal bipyramidal structure, just slightly distorted (as described above) due to the interactions with C_60_. In addition, looking at the Mulliken population and the natural orbital population neither the endo-Li_3_F_2_(D_3h_) nor the C_60_ show an increase or change of electron charges. The C_60_·Li_3_F_2_ HOMO, the main contribution of which is given by C 2p AO’s, is delocalized almost entirely on the fullerene (Figure 4). The fact that the C_60_·Li_3_F_2_ AIE is much lower than C_60_ AIE, 5.64 vs. 7.08 eV, respectively, can be explained using a molecular orbital character argument. The interaction of C_60_ with the superalkali makes the HOMO of fullerene less bonding, and consequently, most of the C-C bonds are elongated by 0.1–0.2 Å. Upon ionization, the C-C bonds in C_60_·Li_3_F_2_ are shortened on average by 0.1 Å, which can be interpreted as the removal of an electron from a HOMO with antibonding character.

**FIGURE 5:**
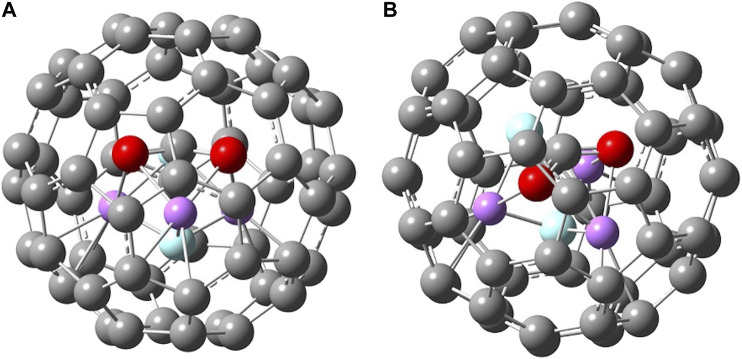
Two different views of B3LYP/6-31G* optimized geometry of C_60_ · Li_3_F_2_(D_3h_) · CO_2_.

**FIGURE 6:**
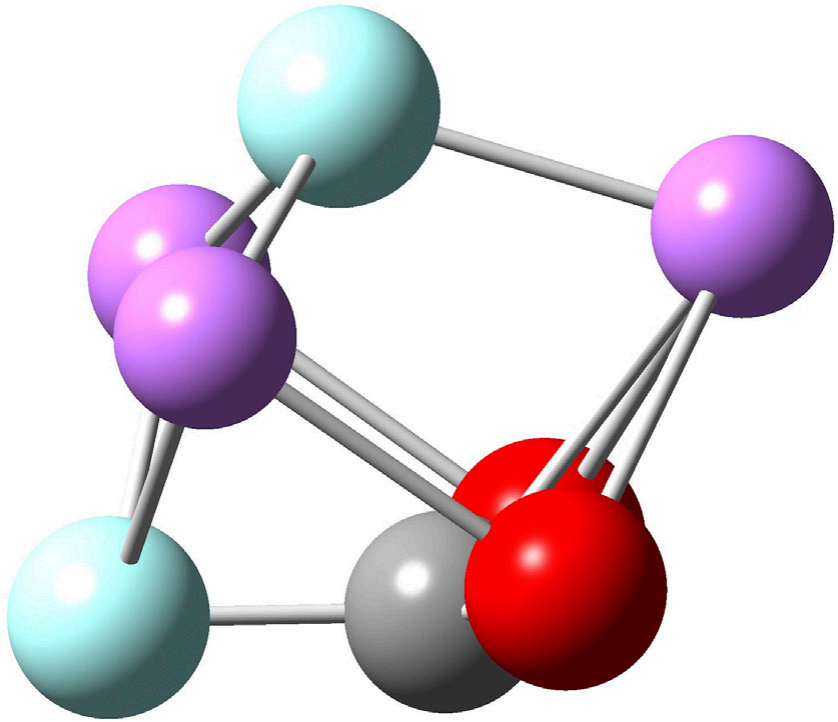
B3LYP/6-31G* optimized geometry of the Li_3_F_2_^.^CO_2_ complex inside C_60_.

The insertion of CO_2_ within C_60_⋅Li_3_F_2_(D_3h_) produces an unexpected result (Figure 5). In previous computational studies, (Zhao et al., 2017; Park and Meloni, 2017; Sikorska and Gaston, 2020), naked superalkali have been shown to be capable of reducing carbon dioxide by transferring an electron and yielding an activated bent CO_2_^−^. In this investigation, we show that our molecular vessel C_60_ forces the reaction to proceed in a different way (Figure 6). From an analysis of the optimized geometries inside fullerene, it is clear that CO_2_ is activated by showing a ∠OCO^°^ bond angle of 132^°^ and C-O bond lengths of 1.20 Å, 0.03 Å longer than r_C-O_ in CO_2_ but 0.06 Å shorter than r_C-O_ in the free Li_3_F_2_(D_3h_)⋅CO_2_ species. The activation of CO_2_ is achieved by a F transfer from Li_3_F_2_ to CO_2_ with the formation of a C-F bond of 1.38 Å, almost identical to the r_C-F_ of 1.382 Å in CH_3_F. (Demaison et al., 1999). This moiety FCO_2_ does not resemble either the fluorocarboxyl radical FCO_2_ for which r_C-O_ is 1.234 Å, r_C-F_ is 1.310 Å, and ∠OCO^°^ bond angle is 118.8^°^, (Zelinger et al., 2003), or the fluoroformate ion FCO_2_^−^ for which r_C-O_ is 1.234 Å, r_C-F_ is 1.46 Å, and ∠OCO^°^ bond angle is 135.9^°^. (Arnold et al., 1995; Thomas et al., 2018). In addition, both fluorocarboxyl radical and fluoroformate ion are planar, whereas the endo-reaction species (Figure 6) resembles a non-planar (trigonal pyramidal) FCO_2_ that interacts with what it looks like a FLi_3_ species. All the attempts to optimize this structure outside C_60_ as free endo-Li_3_F_2_⋅CO_2_ returned a Li_3_F_2_(D_3h_)⋅CO_2_ geometry. Unfortunately, this prevents us from quantifying the interaction of Li_3_F_2_(D_3h_) with CO_2_ inside C_60_. In fact, the reaction we need is

$${\text{C}}_{60}\cdot \left[\text{SA}\cdot {\text{CO}}_{2}\right]+C_{60}\to C_{60}\cdot Li_{3}F_{2}\left(D_{3h}\right)+C_{60}\cdot CO_{2}$$ (1)

from which the interaction of Li_3_F_2_(D_3h_) with CO_2_ can be derived if we were able to find the [SA⋅CO_2_] reaction product as a free species and then its binding energy (or negative solvation energy) with C_60_, i.e.,

$${\text{C}}_{60}\cdot \left[\text{SA}\cdot {\text{CO}}_{2}\right]\to {\text{C}}_{60}+\left[\text{SA}\cdot {\text{CO}}_{2}\right]$$ (2)

In fact, the interaction of Li_3_F_2_(D_3h_) with CO_2_ inside fullerene can be calculated as:

$$\Delta_{r}H^{o}\left(1\right)-BE\left(C_{60}\cdot CO_{2}\right)-BE\left(C_{60}\cdot Li_{2}F_{3}\left(D_{3h}\right)\right)+BE\left(C_{60}\cdot \left[SA\cdot CO_{2}\right]\right)$$ (3)

In other words, this expression tells us that the interaction between endo-Li_3_F_2_(D_3h_) and endo-CO_2_, i.e., the BE of superalkali-CO_2_ in fullerene, is equal to the enthalpy of reaction (1) plus the solvation energies of CO_2_ and Li_3_F_2_(D_3h_) minus the solvation energy of [SA⋅CO_2_]. Because we cannot derive this last solvation energy absolute value due to the impossibility of optimizing the free endo-[SA⋅CO_2_] species, we can estimate this interaction by performing a single-point energy calculation of the free [SA⋅CO_2_] optimized inside C_60_. This structure necessarily represents a higher energy structure than a real minimum and, therefore, the estimated BE of superalkali-CO_2_ in fullerene would denote an upper bound providing us some insights on this interaction. From this computation we get an upper bound for $\text{B}\text{E}\left(C_{60}\cdot \left[SA\cdot CO_{2}\right]\right)$ of −965 kJ mol^−1^, which tells us that this endo-product is highly destabilized by C_60_!

## Conclusion

The activation of CO_2_ by the Li_3_F_2_ superalkali within C_60_ has been investigated at the B3LYP/6-31G* level of theory. C_60_ has been utilized as a reaction vessel and its interaction with the reactants, superalkali and carbon dioxide, have been computed. C_60_ is capable of forcing a superalkali geometry, which does not present the global minimum in the gas phase. Specifically, Li_3_F_2_ takes the D_3h_ structure. C_60_ has a stabilizing effect on the superalkali but a destabilizing effect on the CO_2_, as it can be deduced by the binding energies of these two systems, BE(C_60_⋅CO_2_) = −147 kJ mol^−1^ and BE(C_60_⋅Li_3_F_2_) = 119 kJ mol^−1^. Upon interaction of Li_3_F_2_(D_3h_) with CO_2_ inside fullerene, CO_2_ is clearly activated showing a ∠OCO^°^ bond angle of 132^°^ and C-O bond lengths of 1.20 Å, 0.03 Å longer than r_C-O_ in CO_2_ but 0.06 Å shorter than r_C-O_ in the free Li_3_F_2_(D_3h_)⋅CO_2_ species. The activation of CO_2_ is achieved by a F transfer from Li_3_F_2_ to CO_2_ with the formation of a C-F bond of 1.38 Å. Due to the impossibility of optimizing a free superalkali-CO_2_ complex, [SA⋅CO_2_], resembling the one optimized within the C_60_, a single-point energy calculation has been performed on the free [SA⋅CO_2_]. This energy has been utilized to provide an upper bound for the binding energy of Li_3_F_2_(D_3h_) with CO_2_ within C_60_ of −965 kJ mol^−1^, showing that C_60_ destabilizes the reaction product.

## Data Availability

The original contribution presented in the study are included in the article/Supplementary Material, further inquiries can be directed to the corresponding author.
