# Supplementary material for: CO2 Activation Within a Superalkali-Doped Fullerene
Source: Front Chem. 2021 Jul 14;9:712960. doi: 10.3389/fchem.2021.712960 (PMC8317170; doi:10.3389/fchem.2021.712960)
Supplement: Supplementary file 1 [file DataSheet1.PDF]

Supplementary Material

1 Supplementary Data

Cartesian coordinates of all the B3LYP/6-31G* optimized geometries are presented below.

$Li_3F_2(C_{2v})$

Li	0.00000000	1.02734700	0.00000000
Li	1.51502200	-1.37896300	0.00000000
Li	-1.51511200	-1.37902300	0.00000000
F	1.52617600	0.28846900	0.00000000
F	-1.52614600	0.28841100	0.00000000

$Li_3F_2(C_{2v})$ anion

Li	0.81692700	0.00000100	0.00000000
Li	-1.38038200	-1.59940800	0.00000000
Li	-1.38038200	1.59940300	0.00000000
F	0.32397300	-1.61783200	0.00000000
F	0.32397300	1.61783300	0.00000000

$Li_3F_2(C_{2v})$ cation

Li	0.00000000	0.18608900	0.00000000
Li	3.30565100	-0.19788100	0.00000000
Li	-3.30565000	-0.19789100	0.00000000
F	1.69034800	0.03494800	0.00000000
F	-1.69034800	0.03494600	0.00000000

$Li_3F_2(D_{3h})$

Li	0.00307200	0.81138100	1.11042400
Li	-0.00292300	-1.36806600	0.14738000

Li	-0.00045500	0.55608100	-1.25765000
F	-1.20158900	0.00096600	0.00046700
F	1.20169000	-0.00076500	-0.00051800

$Li_3F_2(D_{3h})$ anion

Li	0.00170100	-1.00074700	-0.94323000
Li	-0.00057400	1.31888300	-0.39294500
Li	-0.00114500	-0.31813800	1.33805800
F	-1.21423900	-0.00028700	-0.00071300
F	1.21424500	0.00028800	0.00008600

$Li_3F_2(D_{3h})$ cation

Li	0.00279600	-1.15660100	-0.79723700
Li	-0.00140500	1.27060600	-0.60240800
Li	-0.00119800	-0.11187000	1.40041600
F	-1.17644700	-0.00104800	-0.00056200
F	1.17638300	0.00033600	0.00030500

$Li_3F_2(C_{2v})CO_2$

Li	2.36459300	0.08350200	0.00000000
Li	0.13215300	-1.86294100	0.00000000
Li	0.00000000	1.86714000	0.00000000
F	1.76595600	-1.49470300	0.00000000
F	1.65590500	1.61565900	0.00000000
C	-2.10257400	-0.07430000	0.00000000
O	-1.64494500	1.08316600	0.00000000
O	-1.56399800	-1.19640600	0.00000000

$Li_3F_2(D_{3h})CO_2$

Li	1.01017700	-0.03113000	-1.15226900
Li	1.01181000	-0.02708900	1.15233400
Li	-0.85144400	1.46696800	-0.00136300
F	1.65383400	-1.10937800	0.00144800
F	0.86192100	1.45221900	-0.00171100
C	-1.34557900	-0.70247100	0.00082800
O	-1.12947400	-0.19536700	-1.12882700
O	-1.13051900	-0.19175700	1.12898900

Endo-$Li_3F_2CO_2$

Li	-0.74784800	-1.37595000	-0.64548900
Li	-0.77536000	1.36422900	-0.63981600
Li	-0.43105600	-0.00938800	1.60619900
F	0.36340400	0.00480600	-1.33287100
F	-1.37742900	-0.01340600	0.16522700
C	0.73671300	0.00803700	-0.00605600
O	0.69036600	-1.09281100	0.47747200
O	0.66628800	1.10626600	0.48034000

C_{60}

C	-0.88157800	0.67025400	3.37263400
C	0.55214000	0.45528600	3.47640300
C	1.43850400	1.38640400	2.93370600
C	0.92824100	2.57142800	2.26453100
C	-0.44830900	2.77767200	2.16457000
C	-1.37147900	1.80781400	2.72970300
C	-1.51403800	-0.61924200	3.15041100
C	-0.47119500	-1.63097100	3.11735300

C	0.80577200	-0.96700200	3.31905100
C	1.93575700	-1.40166300	2.62486200
C	2.61538400	0.93386000	2.21116400
C	1.78984900	2.85125200	1.12795300
C	1.24006700	3.32575700	-0.06375000
C	-0.19354100	3.54023800	-0.16788500
C	-1.02066700	3.27205700	0.92358500
C	-2.29780800	2.60823600	0.72182800
C	-2.51413600	1.70280300	1.83791200
C	-3.12151900	0.46467300	1.62477300
C	-2.61168700	-0.72005900	2.29456500
C	-0.56724300	-2.70329800	2.22951900
C	-1.71034700	-2.80837500	1.33797100
C	-2.71151100	-1.83676000	1.36955700
C	-3.28400100	-1.34216600	0.12855300
C	-3.53763200	0.08022000	0.28637200
C	-3.32937300	0.94927200	-0.78543400
C	-2.69715400	2.23904400	-0.56341400
C	-1.83571000	2.51850500	-1.70023600
C	-0.60937600	3.15573500	-1.50633000
C	2.83218500	1.83887800	1.09479800
C	1.51403800	0.61924100	-3.15041100
C	2.61168700	0.72005800	-2.29456400
C	3.12151800	-0.46467300	-1.62477400
C	2.51413600	-1.70280300	-1.83791400

C	1.37148000	-1.80781500	-2.72970300
C	-0.55214000	-0.45528600	-3.47640300
C	-0.80577200	0.96700300	-3.31905100
C	0.47119400	1.63097100	-3.11735300
C	0.56724200	2.70329800	-2.22951900
C	1.71034800	2.80837500	-1.33797000
C	2.71151000	1.83676000	-1.36955500
C	3.53763100	-0.08022100	-0.28637100
C	3.32937200	-0.94927100	0.78543300
C	2.69715300	-2.23904400	0.56341400
C	2.29780800	-2.60823800	-0.72182800
C	1.02066800	-3.27205800	-0.92358600
C	0.44830900	-2.77767300	-2.16457000
C	-0.92824100	-2.57142800	-2.26453000
C	-1.43850300	-1.38640300	-2.93370500
C	-1.93575800	1.40166400	-2.62486100
C	-2.85875400	0.43191500	-2.05951800
C	-2.61538400	-0.93386000	-2.21116300
C	-2.83218500	-1.83887700	-1.09479800
C	-1.78984800	-2.85125100	-1.12795200
C	-1.24006600	-3.32575700	0.06374900
C	0.19354100	-3.54023900	0.16788600
C	0.60937600	-3.15573600	1.50632900
C	1.83571000	-2.51850500	1.70023500
C	3.28400000	1.34216600	-0.12855300
C	2.85875400	-0.43191500	2.05951800

C	0.88157800	-0.67025400	-3.37263500

$C_{60}CO_2$

C	0.43015900	-0.50807000	-3.47046500
C	1.77980200	-0.13259100	-3.06597200
C	2.55490400	-0.99498300	-2.28470800
C	1.98817000	-2.26877300	-1.86890300
C	0.69070700	-2.62736800	-2.25811100
C	-0.10580900	-1.73172400	-3.07768900
C	-0.40825200	0.67743300	-3.44120100
C	0.42616100	1.78814500	-3.01810100
C	1.77725400	1.29156600	-2.78533700
C	2.54977400	1.79546800	-1.73469300
C	3.37555800	-0.47510300	-1.19181300
C	2.44663200	-2.54021700	-0.51629700
C	1.57163600	-3.15229600	0.38666100
C	0.22296000	-3.52625200	-0.02087200
C	-0.21123300	-3.27127600	-1.31860000
C	-1.56136100	-2.77497500	-1.55468300
C	-1.49541700	-1.82043700	-2.64531600
C	-2.30798200	-0.68398100	-2.62284300
C	-1.74395200	0.59346900	-3.02656000
C	-0.11370000	2.76952300	-2.19082800
C	-1.50337100	2.68314400	-1.75806400
C	-2.31209100	1.62040500	-2.16886800

C	-3.23721800	0.98265500	-1.23437800
C	-3.23469500	-0.45145000	-1.51694200
C	-3.30122800	-1.37477000	-0.46287300
C	-2.43781300	-2.55397900	-0.48768000
C	-1.97931400	-2.81440000	0.86699800
C	-0.68053700	-3.28981500	1.09203400
C	3.30605300	-1.43688300	-0.09133800
C	0.40809500	-0.67742100	3.44108900
C	1.74377300	-0.59346600	3.02642500
C	2.30773400	0.68398500	2.62266700
C	1.49523300	1.82048100	2.64518800
C	0.10564800	1.73178000	3.07757600
C	-1.77988300	0.13259600	3.06572200
C	-1.77737500	-1.29154700	2.78509100
C	-0.42631700	-1.78813500	3.01793200
C	0.11352900	-2.76952900	2.19066300
C	1.50320700	-2.68315800	1.75789600
C	2.31189400	-1.62040400	2.16869800
C	3.23430400	0.45141000	1.51669300
C	3.30082100	1.37468300	0.46263800
C	2.43751500	2.55392300	0.48747900
C	1.56115700	2.77500200	1.55453000
C	0.21106300	3.27133800	1.31845600
C	-0.69085600	2.62741000	2.25796600
C	-1.98826800	2.26874800	1.86867900
C	-2.55495300	0.99494700	2.28442000

C	-2.54994800	-1.79546400	1.73447700
C	-3.37317300	-0.90260000	0.92009000
C	-3.37564600	0.47506800	1.19156600
C	-3.30617500	1.43685200	0.09112500
C	-2.44675000	2.54018500	0.51609100
C	-1.57178000	3.15229700	-0.38683700
C	-0.22311800	3.52628300	0.02071500
C	0.68034800	3.28982900	-1.09219900
C	1.97909800	2.81438800	-0.86718900
C	3.23695200	-0.98266400	1.23416000
C	3.37292000	0.90256100	-0.92030400
C	-0.43029300	0.50809800	3.47032300
C	0.00150400	-0.00069600	0.00190000
O	-1.15066700	0.00053800	-0.01387200
O	1.15362300	0.00002900	0.01665800

$C_{60}Li_3F_2$

C	2.23670100	-1.22793600	2.48382100
C	1.14146800	-0.68447300	3.28757000
C	-0.12788500	-1.31597500	3.28598500
C	-0.34713700	-2.49298600	2.48306400
C	0.70522000	-3.01522900	1.70783100
C	2.01894100	-2.36873900	1.70950700
C	3.02054300	-0.11997400	1.95158400
C	2.38336000	1.10853500	2.41153900
C	1.23218200	0.75535000	3.24574800

C	0.04887700	1.53567100	3.20090400
C	-1.35111700	-0.51314100	3.23680400
C	-1.70501500	-2.42703700	1.94035700
C	-1.96649500	-2.88739400	0.64870700
C	-0.87584300	-3.43440100	-0.15500200
C	0.43956100	-3.49249200	0.37350000
C	1.58773600	-3.13713800	-0.45848700
C	2.57682600	-2.45292700	0.37002600
C	3.32755700	-1.38625000	-0.15249800
C	3.56011700	-0.19398900	0.65658700
C	2.30458500	2.21472100	1.56382900
C	2.86449700	2.14565700	0.22273600
C	3.47702100	0.96586000	-0.22775100
C	3.20026200	0.48562300	-1.57652000
C	3.10871700	-0.96433000	-1.53013100
C	2.15560100	-1.62757700	-2.32539700
C	1.38611700	-2.73796900	-1.78365800
C	0.02370000	-2.67731000	-2.33053100
C	-1.08736900	-3.02220200	-1.52300200
C	-2.33553200	-1.20724400	2.41203500
C	-3.01649800	0.12176000	-1.95021000
C	-3.55565400	0.19553300	-0.65708600
C	-3.32570100	1.38904700	0.15102900
C	-2.57463100	2.45573000	-0.37077400
C	-2.02188600	2.37702200	-1.71484600
C	-1.13749800	0.68608400	-3.28347400

C	-1.22758100	-0.74923500	-3.23766600
C	-2.37874100	-1.10515300	-2.41005800
C	-2.31287400	-2.21771300	-1.56891400
C	-2.86974800	-2.14719100	-0.22478900
C	-3.48079800	-0.96546800	0.22632800
C	-3.10887800	0.96801800	1.52651000
C	-2.15780000	1.63455800	2.32095400
C	-1.38232100	2.73473900	1.77405700
C	-1.58738400	3.13848700	0.45445200
C	-0.44206200	3.49737400	-0.37664900
C	-0.70816700	3.03391200	-1.71809500
C	0.34753200	2.50948400	-2.50122600
C	0.12764000	1.31822100	-3.28843200
C	-0.04986600	-1.52506900	-3.19606500
C	1.25987800	-0.87566100	-3.18919500
C	1.34757700	0.51583500	-3.23433000
C	2.33322900	1.20796300	-2.41634300
C	1.70814600	2.43537500	-1.94918000
C	1.96142900	2.88750800	-0.65124900
C	0.86953000	3.42975800	0.15236500
C	1.07967200	3.01358800	1.51507700
C	-0.02659200	2.67441600	2.31911400
C	-3.20252300	-0.48454400	1.57350400
C	-1.26558800	0.88261200	3.19662500
C	-2.23087500	1.22922900	-2.48194100

Li	0.02245700	-1.29747700	-0.51533500
Li	-0.03243500	0.12420900	1.39064600
Li	0.03081400	1.09058600	-0.80386000
F	-1.09982600	-0.02045600	-0.01530900
F	1.11010400	-0.01259900	0.04572600

$C_{60}Li_3F_2CO_2$

C	-3.18320800	1.12272800	1.34939300
C	-2.18145700	1.92892800	2.07073300
C	-1.43686000	2.93200200	1.41619500
C	-1.65292500	3.16670600	-0.00167400
C	-2.60199300	2.38235000	-0.71994800
C	-3.38986300	1.34011300	-0.02800600
C	-3.10578700	-0.25127200	1.87663400
C	-2.06398100	-0.26671900	2.92351800
C	-1.51680900	1.08523000	3.05475000
C	-0.12575700	1.28099900	3.32883200
C	-0.00656900	3.16435200	1.71620000
C	-0.33705500	3.50652700	-0.59092100
C	-0.01851700	3.00883200	-1.87628100
C	-0.98049700	2.19779500	-2.63113800
C	-2.25132600	1.88407900	-2.05186700
C	-2.78637100	0.53197400	-2.17724300
C	-3.51164700	0.18820200	-0.94198800
C	-3.43929500	-1.12818600	-0.43704700
C	-3.22498900	-1.35263400	1.00418600
C	-1.19511900	-1.38372200	3.05456500

C	-1.31118800	-2.51361200	2.14926500
C	-2.30062100	-2.49314700	1.14323000
C	-1.96631200	-2.99794200	-0.18168100
C	-2.64471300	-2.14198600	-1.16266400
C	-1.96590800	-1.79395900	-2.36025100
C	-2.03686100	-0.43596000	-2.87594300
C	-0.74047800	-0.11301700	-3.50627300
C	-0.22041500	1.19428400	-3.38275500
C	0.67274400	3.54448400	0.47559900
C	3.02741300	0.25286500	-1.84992700
C	3.14449600	1.34390800	-0.96900300
C	3.36643100	1.12707200	0.46038900
C	3.42992700	-0.19334300	0.94957100
C	3.32002700	-1.34359500	0.04888700
C	2.16233600	-1.93275700	-2.07799100
C	1.52655100	-1.09502000	-3.10015800
C	2.06426900	0.26506600	-2.96070100
C	1.21890700	1.39543700	-3.10356800
C	1.33148500	2.50894800	-2.16109500
C	2.28960000	2.51891700	-1.12153700
C	2.65622000	2.18450700	1.19570700
C	2.00291900	1.83765900	2.40409500
C	2.05695000	0.45587600	2.88492500
C	2.77158600	-0.55050500	2.20192800
C	2.27233100	-1.92709700	2.09434100

C	2.61488800	-2.42555500	0.75430200
C	1.69079300	-3.22608300	0.02555900
C	1.46425900	-2.95177600	-1.39437900
C	0.13862400	-1.27898200	-3.37887900
C	-0.60590600	-2.30342300	-2.64229200
C	0.03260900	-3.12802800	-1.68250000
C	-0.64518500	-3.49465700	-0.43917300
C	0.37369900	-3.53927400	0.62135000
C	0.04400500	-3.05032100	1.90254200
C	1.00505300	-2.24499600	2.66204600
C	0.24270300	-1.19688800	3.34428800
C	0.75169300	0.11771500	3.46175900
C	1.98712500	3.05304900	0.20848200
C	0.63794900	2.34093500	2.66424600
C	3.10260300	-1.10960700	-1.32064300
Li	-0.74784800	-1.37595000	-0.64548900
Li	-0.77536000	1.36422900	-0.63981600
Li	-0.43105600	-0.00938800	1.60619900
F	0.36340400	0.00480600	-1.33287100
F	-1.37742900	-0.01340600	0.16522700
C	0.73671300	0.00803700	-0.00605600
O	0.69036600	-1.09281100	0.47747200
O	0.66628800	1.10626600	0.48034000
